# Making Mobile Leaflets: Biomechanical Forces in Atrioventricular Valve Formation

**DOI:** 10.3390/cells15060562

**Published:** 2026-03-20

**Authors:** Anji Yang, Renee Wei-Yan Chow

**Affiliations:** Australian Regenerative Medicine Institute, Monash University, Clayton, VIC 3800, Australia; ayan0049@student.monash.edu

**Keywords:** heart, valve, forces, morphogenesis, development, atrioventricular valve, hemodynamics

## Abstract

Atrioventricular valves prevent the backward flow of blood from the ventricles to the atria and are essential for the efficient pumping of blood throughout the body. Errors in development can lead to congenital atrioventricular valve disease. Atrioventricular valve formation is a multi-step process that involves endocardial cushion formation, valve progenitor cell proliferation, valve sinus formation, valve elongation, and extracellular matrix remodeling. Increasing evidence suggests that hemodynamic cues are required across multiple steps. Here, we compare atrioventricular valve formation in different in vivo models and review how biomechanical forces regulate atrioventricular valve formation.

## 1. Introduction

The adult human heart beats around 100,000 times a day. During each heartbeat, the atrioventricular (AV) valves open and close in response to pressure gradients across the ventricles and atria, ensuring unidirectional blood flow across the chambers.

AV valve development is a complex process that involves a series of overlapping steps, including endocardial cushion formation, valve progenitor cell proliferation, valve sinus formation, valve elongation, and extracellular matrix (ECM) remodeling. Errors during development can cause congenital AV valve disease, where the AV valves either do not close properly, causing blood to leak backward, or do not open fully, causing the heart muscle to work harder to pump blood around the body. Common congenital AV valve diseases include mitral stenosis, mitral insufficiency, tricuspid atresia, tricuspid stenosis, and Ebstein anomaly. Collectively, these diseases occur in about 4 per 10,000 live births [1].

Human embryos with a slow heart rate at a gestational age of 7 weeks have a higher risk of congenital heart defects [2], raising the possibility that abnormal biomechanical forces may play a role. In line with this, over 60% of congenital heart diseases do not have a genetic explanation and are believed to have a multifactorial origin [3]. In this review, we compare AV valve development in mouse, chicken, and zebrafish—three commonly used in vivo models of heart development—and review the evidence on the role of biomechanical forces caused by heartbeat and blood flow in AV valve formation.

## 2. The Adult Atrioventricular Valves

**Figure 1:**
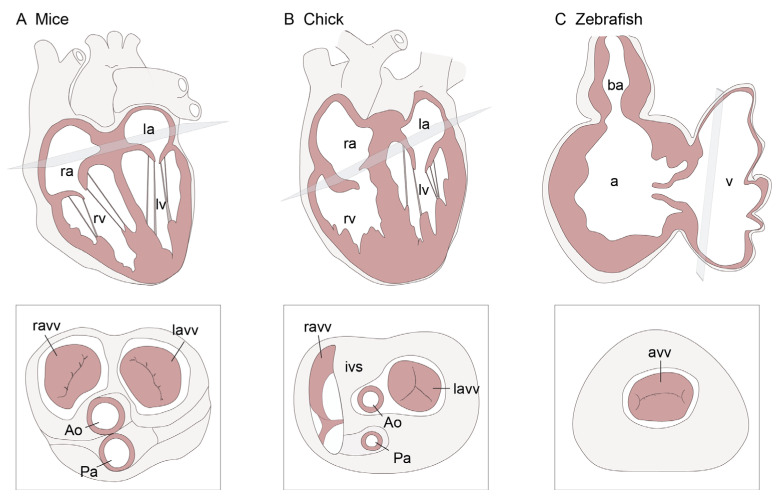
Comparative transverse and frontal heart schematics across mouse, chick, and zebrafish. (**A**) Mouse heart section illustrating the four-chambered heart and AV valves. The AV leaflets are supported by chordae tendineae and papillary muscles; both AV valves in mice are bicuspid. (**B**) Chick heart schematic illustrating the four-chambered structure. In chickens, the left AV valve is composed of three leaflets, whereas the right AV valve consists of a single muscular flap. (**C**) Zebrafish heart section illustrating the two-chambered structure. The AV valve consists of four leaflets. ra, right atrium; la, left atrium; rv, right ventricle; lv, left ventricle; ravv, right atrioventricular valve; lavv, left atrioventricular valve; avv, atrioventricular valve; Ao, aorta; pa, pulmonary artery; ba, bulbus arteriosus; ivs, interventricular septum.

Mice and chickens are classic models for studying heart development and, like humans, both possess four-chambered hearts (Figure 1A,B). Like humans, the left AV valves operate under significantly higher pressure than the right AV valves. In humans, the tricuspid valve is located on the right side and is composed of three leaflets, while the mitral valve is located on the left side of the heart and is composed of two valve leaflets. Mice differ from humans in that the right AV valve is formed from two leaflets [4]. In chickens, the left AV valve has three leaflets instead of two, while the right AV valve consists of a single, muscular flap [5].

In humans and mice, the AV valve leaflets are supported by chordae tendineae and associated papillary muscles, which prevent them from bulging backward into the atria during ventricular systole. The function of the chordae tendineae can be defined more specifically based on their region of attachment to the leaflet. Although the mouse right AV valve is bicuspid, it has been argued to be functionally tricuspid due to the papillary muscle configuration, which supports similar mechanical force transmission to that in humans [4]. In chicks, chordae tendineae and papillary muscles clearly support the left AV valve. Some argue that the right AV valve in chicks attaches directly to the ventricular free wall and interventricular septum [5], while others suggest that there are very short chordae tendineae present [6,7].

The zebrafish is a newer animal model. Zebrafish have a two-chambered heart, and their AV valve is quadricuspid, comprising two major leaflets and two minor leaflets [8,9] (Figure 1C). Intracardiac pressures in adult zebrafish are much lower compared to those in chick and mice, and zebrafish lack chordae tendineae and papillary muscles. The major AV valve leaflets appear to be supported by trabecular bands attached to the base of the valve leaflets [10,11]. These ventricular trabeculae may bear part of the stress generated by cardiac contraction, making them a simplified model for the interaction between the valve and the ventricular wall [12,13].

At the cellular level, AV valves consist of valve interstitial cells (VICs) and ECM components encased by a monolayer of valve endothelial cells [14,15]. In humans, the ECM is clearly stratified into three layers: the collagen-rich fibrosa, the proteoglycan-rich spongiosa, and the elastin-rich ventricularis [15,16,17]. Mouse and chick AV valves share a similar trilaminar microarchitecture, although the layers are less distinct compared to those in humans [16]. By contrast, clear ECM stratification has not been observed in zebrafish AV valves [13,18]

## 3. Valve Morphogenesis

### 3.1. The Formation of the Endocardial Cushions

The formation of AV valves starts shortly after the looping of the primitive heart tube. In mice and chicks, AV valve development begins with the formation of a pair of ECM swellings at the superior and inferior edges of the AV canal (embryonic day 9.5 [E9.5] in mouse; Hamburger–Hamilton stage 14 [HH14] in chick) [19,20,21]. These swellings are called endocardial cushions. Bone morphogenetic proteins secreted by the underlying myocardium activate the AV endocardium, allowing a subset of endocardial cells to undergo endothelial-to-mesenchymal transition (EndoMT) [22]. The newly formed mesenchymal cells further proliferate and contribute to the growth of the cushions.

A second pair of endocardial cushions forms on the lateral sides of the AV canal as the initial pair of cushions begins to fuse and form the AV septum (E13 in mouse, HH26 in chick) [23]. As these lateral cushions become populated by valve mesenchyme, the underlying myocardial shelf grows and extends from the AV myocardium [24].

In zebrafish, superior and inferior cardiac cushion formation occurs around 36 h after fertilization (hpf). The ECM swellings are much less prominent compared to the superior and inferior cushions of mice and chickens. Concurrently, AV endocardial cells become smaller, resulting in the movement of cells towards the AV canal and a local region of high cell density at the AV canal [25]. Starting at around 50 hpf, a subset of endocardial cells overlying the superior endocardial cushion undergoes a partial EndoMT. These cells collectively migrate into the ECM and form a bilayered structure [26,27]. At this point, the endocardial cushions effectively prevent retrograde blood flow across the AV canal [28]. Similar events occur at the inferior cushion about half a day later [29]. It is not known when the lateral cushions form.

### 3.2. Morphing from Cushions into Leaflets

**Figure 2:**
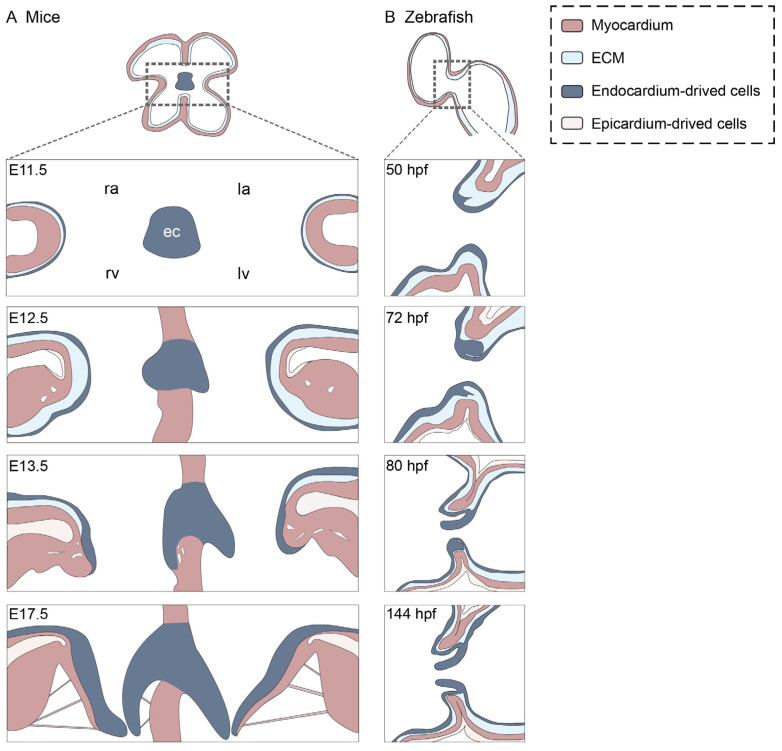
Models of AV valve morphogenesis in mice and zebrafish. (**A**) Valve formation in mouse. At E11.5, the atrioventricular canal contains large mesenchymal cushions that fuse to form the atrioventricular septum. Smaller lateral cushions appear. By E12.5, EndoMT has initiated in the lateral cushions. For the mural leaflets, small gaps start to appear within the myocardium over the developing papillary muscle regions, indicating the start of delamination. At E13.5, the lateral endocardial cushions continue to grow on a myocardial shelf extending from the AV myocardium. The right septal leaflet lacks myocardial support and transitions into a leaflet morphology via elongation. The left septal leaflet elongates while remaining attached to the septum. By E17.5, delamination of the right atrioventricular valve leaflet occurs. (**B**) Valve formation in zebrafish. At 50 hpf, partial EndoMT has initiated at the superior atrioventricular cushion, and some endocardial cells at the ventricular side of the cushion send protrusions into the ECM. At 72 hpf, a subset of endocardial cells has collectively migrated into the ECM at the superior cushion, and collective cell migration is underway in the inferior cushion. By 80 hpf, the superior leaflet has been released through tissue-sheet delamination. The inferior valve remains a cellularized cushion. By 144 hpf, the inferior leaflet has also formed, and both valve leaflets are undergoing elongation. ra, right atrium; la, left atrium; rv, right ventricle; lv, left ventricle; ec, endocardial cushion.

The next stage in AV valve development involves valve sinus formation and the transformation of the cellularized endocardial cushions into free-moving valve leaflets (Figure 2). This and later stages of valve development are poorly studied compared with the earlier stage of endocardial cushion formation.

Studies in mice and chicks suggest that the left septal leaflet in amniotes forms via elongation of the endocardial cushion, driven mainly by cell proliferation, whereas the other leaflets form via delamination (see Box 1). At around E12.5 in mice, or HH26 in chick, mural AV valve leaflets form via fenestrations in the myocardial shelf, which eventually join and allow the leaflets to delaminate and distend into the lumen [30,31]. Some have argued that this process is reliant on the controlled cell death of myocardial cells [24], while others believe that cell death contributes little to the process [32,33,34]. Delamination of the septal leaflet of the right AV valve is delayed relative to the other leaflets, occurring at around E17.5 in mice [24] and HH44 in chick [7]. A study in mice suggests that formation of the right septal leaflet occurs via delamination in the septal myocardium [24]. Meanwhile, a study in chicks suggests that delamination occurs in a region of the endocardial cushion that has been muscularized due to invasion of cardiomyocytes from the septum [7]. More studies are needed to determine which model is correct, or whether this reflects species-specific differences. In chick, but not mice, the right AV valve undergoes myocardialization, a process in which myocardial cells invade the endocardial cushion (HH31) [35]. This muscular nature of the chick’s right AV valve allows it to contract and support right cardiac function [5].

Box 1 **What is delamination?**

The term delamination in the field of heart valve biology can mean different things depending on the context.

**EndoMT**

During early valve development, endocardial cells undergo endothelial-to-mesenchymal transition (EndoMT). During EndoMT, delamination refers to individual cells leaving the endothelial layer and migrating into the extracellular matrix.

**Leaflet formation**

After EndoMT, when endocardial cushions transition into valve leaflets, delamination refers to a tissue-level process where a portion of the valve cushion splits from the cardiac wall or septum to produce a free-moving leaflet.

**Disease and tissue-engineering**

During valve maturation, valve cells produce a highly organized, stratified extracellular matrix. Delamination in heart valve disease or in tissue-engineered heart valves refers to the disruption to the laminar structure of the extracellular matrix.

We recently found that the superior AV valve in zebrafish also forms via delamination. After formation of the bilayer of cells within the ECM, the ECM-embedded cells downregulate vascular endothelial-cadherin (VE-cadherin) while retaining tight junctions to preserve epithelial integrity. The bilayer then repolarises, re-expresses VE-cadherin, and splits, releasing a simple two-cell-thick valve leaflet via mesenchymal–endothelial transition (MEndoT) [28]. Earlier zebrafish work proposed a gradual elongation-based transition occurring over days [18,29]; our time-lapse movies indicate that splitting of the bilayer takes less than an hour. Following delamination, the cells that led the collective migration of endocardial cells into the ECM proliferate at the base of the newly formed leaflets and give rise to cells that migrate into the valve interstitial space, undergo further proliferation, and differentiate into VICs [28]. We propose that the unique microenvironment at the base of the valve acts as a stem cell niche and continuously provides the growing valve with new valve interstitial cell progenitors. The inferior zebrafish AV valve has not been studied in as much detail, but it is likely formed in a similar manner to the superior AV valve about half a day later. It is not known when the lateral valve leaflets form, but they are present in adults [9,11].

Given that zebrafish valve delamination involves MEndoT, we wonder if MEndoT also occurs during amniotic AV valve development. Although amniote delamination occurs within the myocardial layer or within the myocardialised endocardial cushion, mature amniote valves maintain a continuous endothelial lining. The origin of the endothelial cells contributing to the endothelial lining remains unclear. MEndoT has been observed in the context of human and mouse calcific aortic valve disease [36], showing at least that mammalian VICs have the capability of undergoing MEndoT.

In mice and chickens, chordae tendineae start to form during delamination and continue to mature afterwards. Immediately following delamination, muscle cells remain on the surface of newly formed valve leaflets and thin strands of muscle remain attached to the valve leaflet. Over time, the muscle cells on the surface of the leaflet undergo apoptosis and are replaced by valve endothelial cells [24,37]. How chordae tendineae form is not clearly defined. Various studies have shown that chordae tendineae are derived from mesenchymal cells of the endocardial cushions rather than myocardial cells [31,37,38,39].

Zebrafish do not develop chordae tendineae [13]. Instead, disorganised trabecular bands that are attached to the base of the AV valves first appear around 104 hpf and become progressively more defined as the heart matures into adulthood [10].

### 3.3. Valve Elongation, Thinning, and Extracellular Matrix Remodelling

The final step in valve development involves elongation and thinning of the valve leaflets together with extracellular matrix remodelling. There is no significant apoptosis during this step [40,41]. In mice and chickens, the endocardial cushions begin to undergo elongation (E12.5 in mouse, HH30 in chick) even before valve leaflets fully delaminate. At the cellular level, this involves increased cell proliferation at the distal ends of the mitral and tricuspid valve primordia [7,37]. As development proceeds into late gestation, the dispersed population of mesenchymal cells condenses into a denser cellular structure (E15.5 in mouse, HH36 in chick), accompanied by increased cell–cell adhesion and upregulation of matrix-associated markers [7,37,40,41]. This condensation appears first on the atrial side of the leaflet and then extends toward the ventricular side [40,42,43]. During valve condensation, ECM regulatory molecule expression is restricted to create distinct cellular compartments within the leaflets, allowing for the formation of organized connective tissue [37]. As the valve leaflets approach their adult length, cell proliferation decreases. Concurrently, the ECM starts to stratify (postnatal day 7 in mouse, HH42 in chick) [40,44]. Heart valve ECM stratification continues into juvenile stages to form a trilaminar structure [40,41].

In both mice and chicks, in addition to the mesenchyme produced by EndoMT, epicardium-derived mesenchymal cells and neural crest-derived mesenchymal cells also contribute to the valve interstitial cell populations of mature AV valve leaflets [45,46,47]. Studies in mice also suggest a contribution from bone marrow-derived cells. The specific functions of these varied cell populations remain unclear [48].

In zebrafish, the superior and inferior valve leaflets elongate until 90 days after fertilization (dpf) [18]. Cell proliferation occurs mainly at the distal tip of the valves [49]. Lineage tracing suggests assimilation of neural crest-derived cells into the VICs after 10 dpf. However, as in mice and chicks, neural crest-derived cells do not contribute significantly to the adult AV valve [18]. The zebrafish valve undergoes significant remodeling as the valve elongates. Specifically, the valve ECM switches from a promigratory ECM enriched in fibronectin and hyaluronic acid to a pliable ECM rich in Elastin1 from 20 dpf, combined with changes in cell density and morphology as development proceeds [13,18]. Despite the highly regenerative properties of zebrafish, valve stenosis occurs naturally in aged zebrafish [9]. AV valves of aged fish have disordered ECM and significantly higher levels of elastin compared to those of young adult fish, similar to what has been clinically reported for myxomatous valve disease [9]. Thus, although zebrafish AV valves do not show ECM stratification, zebrafish can be a useful model to study ECM remodeling and degeneration. Key differences between the AV valve developmental programs for each species are summarized in Table 1.

## 4. The Role of Biomechanical Cues

The heart continually pumps blood while growing and remodeling, and it has long been suggested that hemodynamic stresses within the heart may provide morphogenic cues to guide valve development [50]. This hypothesis is supported by many excellent in vitro studies showing that both endothelial cells and VICs respond to wall shear stress, tensile stress, and compressive stress. However, determining the role of biomechanical forces in AV valve development requires us to accurately quantify in vivo biomechanical forces in the heart, perturb these forces while causing minimal changes to chemical cues, and measure the valve cell response. To this end, work has been done mainly in three animal models: chicken, mouse, and zebrafish.

### 4.1. Chicken

Early work on the role of biomechanical forces in the heart was predominantly conducted in chicken. The accessibility of the chick embryo allowed for direct visualization and manipulation of the developing heart. Blood flow patterns were first visualized by injecting Indian ink into the bloodstream [51] and biomechanical forces were altered via surgical interventions such as vitelline vein ligation [51,52], left atrial ligation [53,54], and outflow tract banding [53]. These seminal studies helped to establish the importance of biomechanical forces in AV valve development.

More recent studies in chicken have used static images to perform computational fluid dynamics modeling and study hemodynamics in the developing chick heart [55,56], and have combined outflow tract banding or vitelline vein ligation with modern molecular biology techniques to assess the effect of altered hemodynamics on AV valve morphogenesis [57,58]. These studies have shown that patterns of wall shear stress correlate with the expression of valve-specific genes, and that perturbation of biomechanical forces modifies the expression of the mechanosensitive genes TGF-β receptor III, KLF2, and YAP, as well as various ECM proteins [57,59,60]. Interestingly, outflow tract banding also caused a decrease in apoptotic cells located in the right septal endocardial cushion, suggesting a possible role for biomechanical forces in regulating delamination [57].

### 4.2. Mouse

Mice are highly amenable to genetic manipulation and are the principal model used to identify genes involved in heart development. The similarity between mouse heart anatomy and that of humans makes it indispensable for dissecting the genetic components of mechanosensitive signaling pathways during AV valve development. However, inherent challenges in imaging and manipulating the mouse embryo have led to comparatively fewer studies using mice to investigate the role of biomechanical forces. To date, quantitative measurements and computational simulations of the forces acting on developing mouse AV valves remain limited. Several heart contractility mutants, including *Cacnb2* [61] and *Titin* [62], have been used to alter cardiac forces. However, these mutants die by E11.5 or earlier, limiting their use for studying later stages of AV valve development. More recently, transient pharmacological approaches have been used to manipulate embryonic hemodynamics without directly altering gene function. For example, pregnant mice gavaged with a single dose of the class III antiarrhythmic drug dofetilide at 2 mg/kg stops blood flow in E9.5 embryos for a few hours, with complete recovery by 5 h after treatment [63]. This approach provides a reversible method to perturb biomechanical forces and can likely be applied during late stages of valve development. It may also model clinically relevant situations in which maternal medications may transiently alter fetal hemodynamics [63].

**Figure 3:**
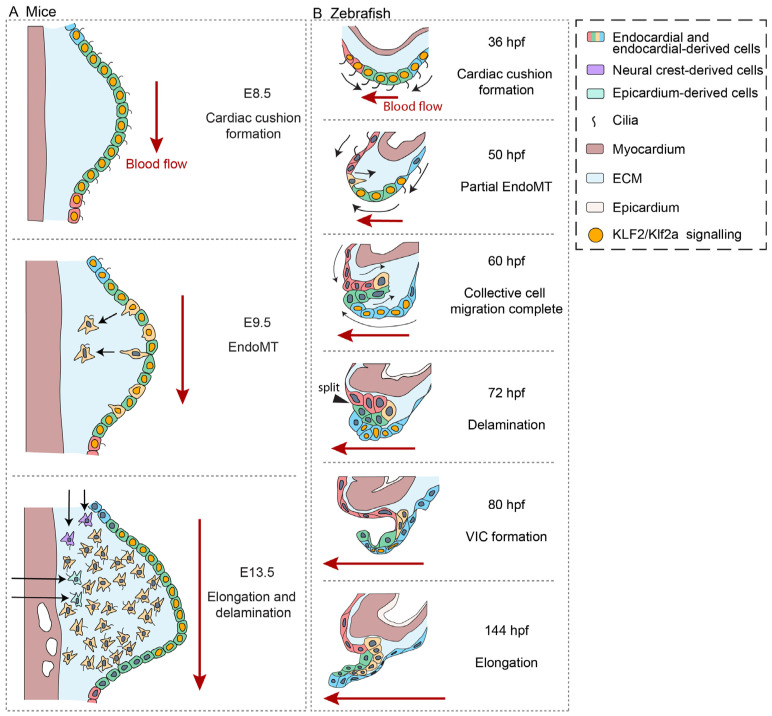
Cilia and KLF2/Klf2a signaling: An example of how wall shear stress regulates cell differentiation in the developing AV valve in mice and zebrafish. (**A**) Early valve development in mouse. At E8.5, endocardial cells of the AV cushion are ciliated. Wall shear stress activates KLF2 signaling (orange nuclei) in endocardial cells of the AV cushion. Cilia-dependent KLF4 signaling inhibits EndoMT at the edges of the cushion. At E9.5, endocardial cells experiencing high wall shear stress at the central region of the AV cushion undergo cilia disassembly, allowing EndoMT to proceed. At E13.5, KLF2 signaling is highest in endocardial cells experiencing high wall shear stress, and mediates valve delamination/elongation. Cells are colored to indicate position and fate. (**B**) Valve leaflet formation in zebrafish. At 36 hpf, cardiac cushion formation occurs concurrently with cells moving towards the AV canal. High wall shear stress, including high oscillatory wall shear stress, activates Klf2a signaling (orange nuclei) in the AV endocardium. At 50 hpf, endocardial cells experiencing high wall shear stress at the central region of the AV cushion undergo cilia disassembly. Klf2a is restricted to the central and atrial sides of the AV cushion and regulates the migration of cells at the ventricular edge into the ECM. Klf2a signaling is required for proper valve delamination at 72 hpf. Klf2 remains present at 80 hpf, when cells at the base of the valve leaflet proliferate to form the first population of VICs. It is not clear if Klf2a signaling persists at 144 hpf during elongation stages. Cells are colored to indicate position and fate. Black arrows represent the direction of cell migration.

Key mechanosensitive genes, including KLF, WNT, NOTCH, NFAT, and YAP, are expressed during AV valve development in mice [22,41,60,64,65]. Endocardial Klf2 deficiency results in loss of Wnt9b expression and reduced canonical WNT signaling in neighboring mesenchymal cells, leading to defective AV valve formation [41]. Another KLF transcription factor, KLF4, is expressed at the ends of the AV canal cushion at E9.5, where it inhibits EndoMT. Interestingly, its expression pattern is regulated by mechanosensitive primary cilia. In the middle of the endocardial cushion, where wall shear stress is the highest, endocardial cells lose cilia, resulting in KLF4 downregulation and allowing EndoMT to progress [66] (Figure 3A).

Loss of NOTCH signaling abrogates EndoMT, while ectopic endocardial Notch1 intracellular domain expression leads to partial EndoMT of ventricular endocardial cells outside the AV canal [22,65]. NOTCH1 is expressed uniformly throughout the endocardium during the initial stages of AV valve formation. By transiently blocking the heartbeat of E9.5 mouse embryos, it was found that the reduced expression of Dll4 in the endocardium led to a ligand-depleted field, enabling NOTCH to be specifically activated in AV canal by wall shear stress [63]. Fascinatingly, Notch activation may depend on cell surface caveolae, which appear localized to areas of increased wall shear stress [63].

NFATc1 is expressed in valve endocardial cells at both EndoMT and post-EndoMT stages [64,67]. NFATc3 and NFATc4 are believed to be active in the mouse myocardium, where they regulate the overlying endocardium by repressing VEGF signaling [67]. Mouse valve endocardial cells marked by the Nfatc1 enhancer do not undergo EndoMT and remain within the endocardium as a proliferative population to support valve elongation [68]. This occurs at least partly because NFATc1 suppresses the expression of the transcription factors *Snail1* and *Snail2*, which are required for the initiation of EndoMT [68]. Disruption of the *Nfatc1* gene results in underdeveloped AV valves, and *Nfatc1* null mutants die around E13.5 [64]. Mutants lacking endocardial calcineurin have smaller, blunter AV valve leaflets than controls at E13.5, a phenotype that is replicated by injecting the calcineurin inhibitor cyclosporine into pregnant mothers at E11, but not when injected at E10 or E12 [67].

YAP has been proposed to be involved in both EndoMT and mesenchymal cell proliferation, and loss of YAP leads to underpopulated AV cushions at E9.5 [69]. The early embryonic lethality of Yap mutants has prevented in vivo study of the effects of Yap on later valve morphogenesis, but YAP is known to be expressed in both valve endothelial cells and VICs until at least E17.5 [60].

### 4.3. Zebrafish

Significant strides in understanding the role of biomechanical forces in AV valve development have been made using the zebrafish model. Zebrafish embryos are fertilized externally and have high transparency, enabling live imaging, image-based force modeling, and microsurgical perturbations of blood flow. Because embryos acquire sufficient oxygen from the water by passive diffusion to support development, zebrafish lacking cardiac contraction can survive to at least 7 dpf, beyond the valve delamination stages of superior AV valve development [70], and zebrafish lacking red blood cells can survive until at least 10 dpf [71], when superior and inferior AV valve leaflets are elongating. There is a range of mutants that affect cardiac contraction [70,72], as well as a recent Tnnt2a degradation line that allows stage-specific control of cardiac contraction [73].

An early study showed that obstructing flow with intracardiac microbeads blocks the initiation of AV valve development [74]. Since then, there have been attempts to distinguish the roles of shear stress, blood flow patterns, strain, pressure, and myocardial activity in AV valve development—a difficult task, as these variables are mechanically coupled. So far, studies have mostly illustrated the importance of wall shear stress in AV valve development. 2D + time simulations suggest that the spatial variation of oscillatory wall shear stress along the AV canal in 48 hpf embryos is important for initiating AV valve formation [75]. In line with the delayed development of the inferior valve leaflet, both unidirectional wall shear stress and oscillatory wall shear stress are lower in the inferior cushion than in the superior cushion at 48 hpf [75]. Blood cells flowing past the endocardial wall increase wall shear stress locally. At 65 hpf, 3D + time simulations combined with particle-tracking velocimetry showed that maximal wall shear stress at the AV canal was approximately 3–4 times lower in *gata1* mutant fish, which lack blood cells, compared to controls. *Gata1* mutants exhibit a non-penetrant, disorganized endocardial cushion phenotype and often have errors in valve delamination, resulting in thickened valves at 4 dpf [28]. The thick valve phenotype in *gata1* mutants can be partially rescued by injecting a viscous medium into the bloodstream, supporting the hypothesis that wall shear stress regulates valve delamination [28]. Consistently, inserting a ferromagnetic bead into the heart and manipulating the bead using magnetic tweezers suggests that shear stress, rather than pressure, leads to mechanosensitive Ca^2+^ influxes [76].

Various force-sensitive ion channels, including TRP channels, PKD channels, and Piezo channels, are involved in zebrafish AV valve development [75,76,77]. These ion channels regulate the expression of *klf2a* and/or *klf2b* in the AV endocardium [75,76,77] (Figure 3B). Klf2a/b is important for both initial cell migration into the ECM and delamination [28,78]. Recent studies have provided insight into how Klf2a intersects with other mechanosensitive pathways to regulate endocardial cell migration into the cardiac jelly. Unlike mouse, where NOTCH1 is expressed uniformly throughout the endocardium during the initial stages of AV valve formation, Klf2a and Notch in zebrafish are expressed in cells of the AV endocardium adjacent to the cells that ingress. Lateral inhibition between endocardial cells, mediated by Notch, singles out Delta-like-4-positive endocardial cells and defines the site at which endocardial cells migrate into the cardiac jelly. The migration of Delta-like-4-positive endocardial cells is dependent on Wnt9a, which is produced in parallel through an Erk5-Klf2-Wnt9a signaling cascade [79,80]. Like mice, immotile cilia have also been proposed to play a role in zebrafish valve development by suppressing valve EndoMT. The number of ciliated cells at the zebrafish AV canal decreases rapidly between 32 and 54 hpf. Given that primary cilia disassemble at ~15 dyn cm^−2^, the timing of cilia loss is consistent with quantitative analyses of wall shear stress showing that cells located at the center of the AV canal during these stages experience an average of ~30 dyn cm^−2^, while those located at the edge of the cushions experience an average of ~10 dyn cm^−2^ [66].

Another major mechanotransduction pathway is the Ca^2+^/calcineurin/Nfatc1 signaling pathway. With the aid of software to phase-match movies of the beating heart [81], biomechanical forces have been shown to activate calcium oscillations in the AV endocardium, which drive Nfat activity [76] These Ca^2+^ oscillations depend on the presence of ATP and purinergic receptors. Unlike mouse, Nfat activity is observed only in the endocardium. Ablating Nfatc1-expressing cells in adult zebrafish results in the death of VICs [82], suggesting that Nfatc1 expression switches from the endocardium to the VICs, or that Nfat is expressed in VICs during development but has non-canonical functions outside the nucleus. Pharmacologically inhibiting calcineurin, a phosphatase whose activity is required for Nfat to migrate into the nucleus, results in different phenotypes when performed at different developmental stages. Treatment from 48 hpf prevents endocardial cell migration into the cardiac jelly and results in underdeveloped valves [76], while pharmacologically inhibiting calcineurin from 60 hpf prevents proper valve delamination and results in thick, hyperplastic valve leaflets [28]. Mosaically inhibiting Nfat activity in Nfat-expressing cells using a genetically encoded Nfat-specific peptide inhibitor promotes their migration into the cardiac jelly and results in disorganized endocardial cushions [76]. Nfatc1 mutants do not show early endocardial cell migration defects and develop abnormally thin valves [18]. More studies are needed to explain the varied phenotypes associated with perturbation of Nfat signaling in zebrafish, including investigations into the roles of Nfats other than Nfatc1.

Recently, mechanical forces have been found to regulate the expression of the transcription factor Egr3 [83]. *Egr3* is expressed in cardiac valve endocardium and AV mesenchyme from collective endocardial cell migration (48 hpf) through to post-delamination stages (96 hpf). Egr3 mutants display normal AV canal patterning, but valve endothelial cells fail to migrate into the cardiac jelly [83]. Unlike Klf2 mutants, the Egr3 phenotype is highly penetrant and resembles the valve-less phenotype of embryos with complete loss of blood flow.

By injecting a microbead into the heart, it has been shown that ectopic forces in the atrium and ventricle can activate valve development gene programs, including *nfat* and *egr3*, outside of the AV canal [76,83]. Given this, one may wonder whether mechanical force lies upstream of all biochemical signaling pathways important for AV valve development. However, an early study has shown that the expression of *bmp-4* in the myocardium, which is critical for AV valve development [84], appears unchanged in silent heart mutants that lack a heartbeat [85]. This suggests that both force-dependent and force-independent mechanisms instruct AV valve morphogenesis.

## 5. Outlook

Accurate descriptions of normal biomechanical forces during each stage of heart development, and an understanding of how altering forces at different stages of heart development affects morphogenesis, are critical for improving in utero surgical interventions for congenital heart disease, as well as for making informed decisions about taking medications that affect cardiac function in the developing embryo. The collective work in chicken, zebrafish, and mouse has made significant inroads into understanding how biomechanical forces influence AV valve development, particularly EndoMT. More studies are needed to understand the role of forces during later stages of valve development. Future research that extends our understanding to later stages of valve development may incorporate work in newer animal models, such as *Danionella cerebrum*, which is transparent even in adulthood [86].

Since the same mechanosensitive ion channels and genes are used at multiple stages of AV valve development, and their disruption at different stages of development can lead to vastly different phenotypes, more work is needed to determine how these genes and ion channels modify the cell behaviors underlying valve morphogenesis. Work on mechanosensitive ion channels will undoubtedly be aided by tools such as GenEpi, a recently developed genetically encoded fluorescent reporter of Piezo1-dependent activity [87]. Future work may involve the examination of auxiliary subunits of mechanosensitive ion channels [88], which could potentially modify the gating kinetics of the ion channels at different stages of valve development.

Micropipette aspiration experiments have shown that chick AV valves increase in stiffness from HH25 to HH34, with the stiffness almost doubling every 24 h [89]. Further studies are needed to integrate the roles of forces caused by blood flow and cardiac muscle contraction with those caused by changes in the ECM and tissue stiffness. This may involve improving microscopes and imaging protocols to the point where genetically encoded tension sensors can be used to visualize morphogenesis [90,91,92,93]. In vitro studies suggest that YAP signaling is both activated by increased valve ECM stiffness and prevents an increase in valve stiffness [60,94]. It will be interesting to determine if the increase in valve stiffness involves the inactivation of YAP in vivo.

Zebrafish continues to lead the way in teasing apart the roles of shear stress, blood flow patterns, strains, pressures, and myocardial activity in AV valve development. While much of the work has focused on the role of wall shear stress dynamics, motion tracking and image-based finite element modelling have recently been used to model strains and tissue stresses in the developing zebrafish ventricle [17,95]. A similar approach applied to the AV valves will yield valuable insights, especially on the role of strains and stresses on valve abluminal cell and AV myocardial cell populations. Computational fluid dynamics simulations and analysis of reporter line expression are often performed in separate embryos, and this can cause difficulties in correlating biomechanical force indices to patterns of gene expression. In addition to the development of new zebrafish lines, these problems may soon be ameliorated through the use of artificial intelligence to segment images and identify morphological landmarks. An ambitious goal would be to merge biomechanical force data with anatomical data and omics data to create comprehensive atlases of zebrafish heart development.

We expect to see significant improvements in image-based force modeling methods in both developing chicken and mouse hearts in the near future. One of the major hurdles in chicken has been the lack of transgenic lines that label the endocardium and myocardium. Making transgenic lines in chicken is challenging, but with the arrival of CRISPR/Cas9 gene editing [96], the development of efficient in vitro culture systems for primordial germ cells [97], and new methods for direct gene delivery [98], easily accessible pipelines for making chicken transgenics are on their way. Recent advances in imaging technology and embryo culture have already enabled live imaging of E7.5 mouse heart [99,100]. Further improvements will enable live imaging of the developing heart at AV valve developmental stages.

## 6. Conclusions

AV valve development provides a striking example of morphogenesis under mechanical load. The differences in AV valve formation across mouse, chick, and zebrafish, as well as differences in valve leaflet formation within the same heart, show that valve formation does not follow a single conserved pathway. Instead, valve formation is regulated by a family of developmental programs.

Despite differences in AV valve development across and within species, a conserved mechanosensitive logic emerges. Mechanical forces are interpreted through force-sensitive ion channels, primary cilia, and mechanotransductive pathways, including KLF, NOTCH, and NFAT signaling. These pathways do not act in isolation but intersect to spatially and temporally regulate cell behaviors such as migration, proliferation, and differentiation. Importantly, mechanical signals do not replace biochemical patterning cues such as BMP signaling; rather, current evidence supports a hybrid model in which genetic pre-patterning defines valve-competent territories, while hemodynamic forces refine cell fate decisions and sculpt tissue architecture within these domains. Emerging evidence suggests that dysregulation of these mechanosensitive pathways may contribute not only to congenital valve defects but also to adaptive and degenerative valve diseases.

Uncovering the precise roles of biomechanical forces during valve development is an exciting and rapidly advancing area of research. Advances in CRISPR/Cas9 are poised to overcome the lack of transgenics in chicks, enabling their use for deeper mechanistic studies, while improvements in embryo culture and live imaging are making later stages of mouse heart development accessible. Meanwhile, zebrafish provides a powerful system for integrating live imaging and mechanical perturbations with emerging tools such as GenEPi transgenic reporters that enable monitoring of Piezo-dependent activity and image-based finite element modelling approaches that map tissue stresses during valve morphogenesis.

## Figures and Tables

**Table 1:** Differences between the AV valve developmental programs in mouse, chick, and zebrafish. Information from zebrafish is based mainly on the development of the superior AV valve, as the development of the other leaflets is not well studied.

Developmental Step	Mouse	Chick	Zebrafish
Endocardial cushion cellularization	EndoMT	EndoMT; Right AV valve undergoes myocardialisation	Partial EndoMT
Superior and inferior endocardial cushions fuse	Occurs	Occurs	Does not occur
Transformation into valve leaflets	Occurs via elongation or delamination within the myocardium	Occurs via elongation, delamination within the myocardium, or delamination within the muscularized endocardial cushion	Occurs via delamination between two layers of endocardial-derived cells
Supporting apparatus formation	Chordae tendineae form as cushions transform into leaflets	Chordae tendineae supporting the left AV valve form as cushions transform into leaflets. Chordae tendineae supporting the right AV valve either do not form, or very short chordae tendineae form.	No chordae tendineae form, trabecular bands form as the valves elongate
Extracellular matrix remodelling	Results in clear stratification of the ECM	Remodeling in the left AV valve results in stratification of the ECM. The right AV valve consists mainly of muscle without clear ECM stratification.	No clear ECM stratification observed

## Data Availability

No new data were created or analyzed in this study.

## Abbreviations

The following abbreviations are used in this manuscript:

AV	Atrioventricular
dpf	Days post-fertilization
EndoMT	Endothelial-mesenchymal transition
ECM	Extracellular matrix
E	Embryonic day
HH	Hamburger–Hamilton stages
Hpf	Hours post fertilization
MEndoT	Mesenchymal-endothelial transition
VIC	Valve interstitial cell
